# Cross-NASA divisional relevance of an Ice Giant mission

**DOI:** 10.1098/rsta.2020.0222

**Published:** 2020-11-09

**Authors:** Ian J. Cohen, Abigal M. Rymer

**Affiliations:** The Johns Hopkins University Applied Physics Laboratory, 11000 Johns Hopkins Road, Laurel, MD 20723, USA

**Keywords:** Ice Giants, Uranus and Neptune, robotic space exploration, cross-disciplinary space science, future missions, NASA science

## Abstract

Robotic space exploration to the outer solar system is difficult and expensive and the space science community works inventively and collaboratively to maximize the scientific return of missions. A mission to either of our solar system Ice Giants, Uranus and Neptune, will provide numerous opportunities to address high-level science objectives relevant to multiple disciplines and deliberate cross-disciplinary mission planning should ideally be woven in from the start. In this review, we recount past successes as well as (NASA-focused) challenges in performing cross-disciplinary science from robotic space exploration missions and detail the opportunities for broad-reaching science objectives from potential future missions to the Ice Giants.

This article is part of a discussion meeting issue ‘Future exploration of ice giant systems’.

## 1. Introduction

Robotic space exploration presents unbridled opportunities to expand human understanding of the natural order of our planet, our solar system, our universe and beyond. Large-scale missions comprise more than just ground-breaking science [1], they also fuel innovation, public engagement and global partnership in ways that few other endeavours can. Deep-space missions that venture into the outer solar system—such as Galileo at Jupiter, Cassini at Saturn and, it is hoped, soon future missions to the Ice Giant planets of Uranus and Neptune—advance astrophysics, planetary science, solar system studies, sociology (e.g. studying team dynamics through multigenerational missions), philosophy and beyond. To truly facilitate this scope of influence and capability requires collaborations that transcend boundaries that sometimes exist due to tradition or funding lines.

NASA funding of robotic space missions comes from the Science Mission Directorate (SMD) and is predominantly supported by one of four divisions: Astrophysics, Earth Science, Heliophysics or Planetary Science. Astrophysics focuses on the origin of the universe and extra-solar system celestial bodies and structures (stars, galaxies, supernovae etc.); Earth Science on interconnected terrestrial processes including weather, atmospheric dynamics, geophysics, hydrology and climate change; Heliophysics on solar and space plasma physics, including space weather, planetary magnetospheric dynamics and aeronomy, and the interaction of our solar system with the local interstellar medium (LISM); and Planetary Science on the formation and characterization of all planetary bodies (from the smallest to the largest) within our solar system and beyond. Obviously, there are large overlaps and synergies across the Divisions. Our emerging expertise in studying exoplanets (planets orbiting stars besides our own), for example, is spear-headed by Astrophysics (remote sensing of exoplanets with large space-based telescopes) with needed expertise also shared across Planetary Science (characterization of exoplanets in the context of the worlds in our own solar system) and Heliophysics (characterization of planet-stellar wind interactions in our habitable astrosphere).

The Divisions within NASA's SMD fund missions and programs based on strategic and/or Congressionally-directed programs as outlined by implementation plans, strategic plans and science plans that are regularly updated at both the SMD and Division levels. The respective science communities outline their priorities in the form of ‘Decadal Survey’ reports administered by the National Academy of Sciences. These Decadal Surveys, generated with broad community input, occur every 10 years and outline the successes, challenges and future vision within each discipline, as defined by the community. The Decadal Surveys are key cornerstones for the community that define the field's vision for the near-term future; however, while they provide references for NASA (and the National Science Foundation), and policymakers, they are *not* guiding documents, nor are the Divisions bound (nor at times able) to follow them completely. Though this review focuses primarily on NASA and the funding processes within the USA, it should be noted that the Science Programme at ESA is likewise guided by community-driven long-term plans that occur roughly each decade and place high value in cross-disciplinary themes [2]. Furthermore, it should be emphasized that most major future space exploration missions (not just those to the Ice Giants) will benefit greatly from shared opportunities and coordination between international agencies, which generally have similar or complementary strategic science goals.

In this review, we recount past successes as well as challenges in performing cross-disciplinary science from robotic space exploration missions and specifically detail the opportunities for broad-reaching science objectives from potential future missions to the Ice Giants.

## 2. Past successes for cross-disciplinary science

**Figure 1.**
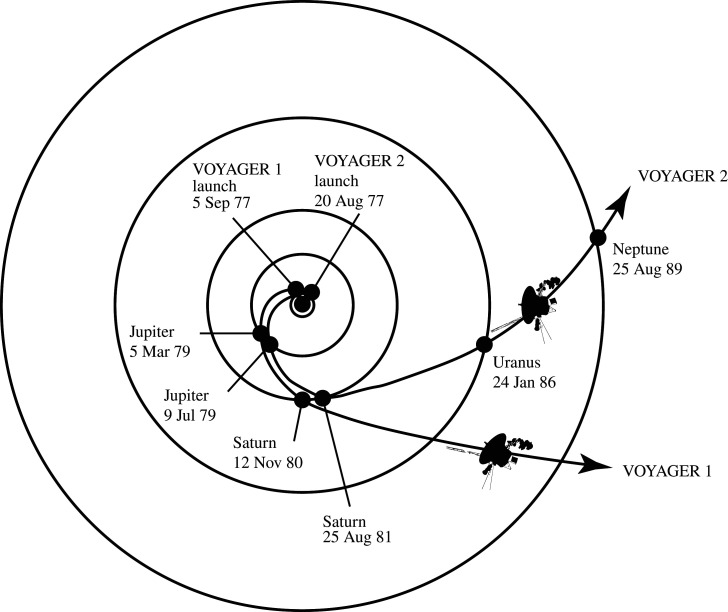
The Voyager Mission, which visited all of the Giant planets, was an ambitious endeavour and became an exemplar of what cross-disciplinary missions could achieve. Credit: NASA/JPL.

**Figure 2.**
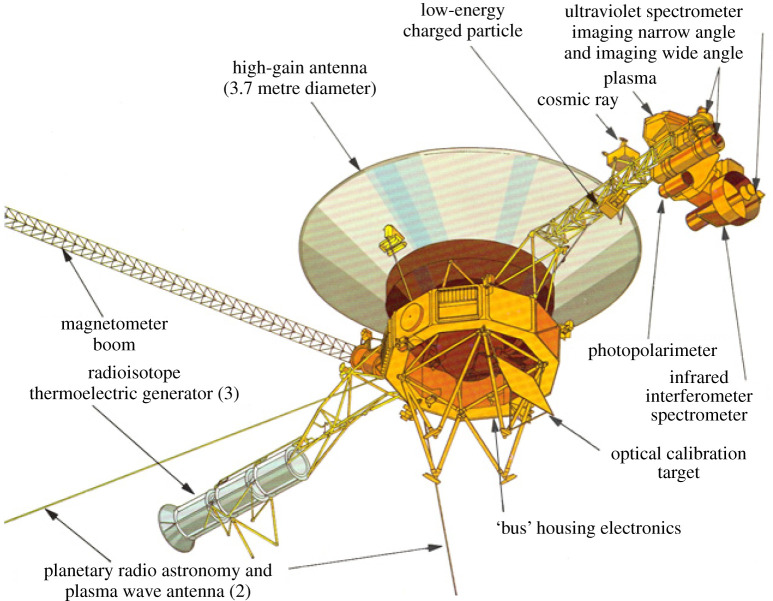
Voyager's ability to provide revolutionary Heliophysics and Astrophysics measurements from beyond the solar system was enabled by its comprehensive *in situ* and remote sensing payload. Credit: NASA/JPL. (Online version in colour.)

**Figure 3.**
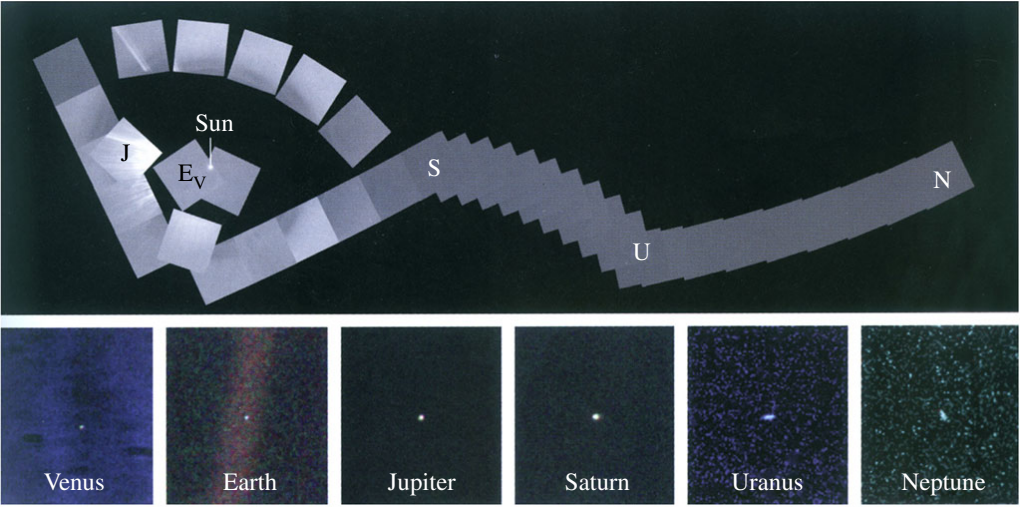
Voyager's visits to the Gas Giants provided humanity its first views of the Gas Giants, and put Earth and the other planets into cosmic perspective. Image credit: NASA/JPL-Caltech. (Online version in colour.)

Perhaps the greatest example of cross-divisional science from a robotic mission comes from the Voyager mission. Voyager's ‘Grand Tour’ of the outer solar system was borne of the realization of a once-in-a-lifetime cosmic alignment of the outer planets in the late twentieth century (figure 1). With the ambitious aim to successfully rendezvous with all four of the Giant planets, the dual Voyager spacecraft (hereafter denoted as ‘V1’ and ‘V2’) were simultaneous developed in the 1970s and instrumented with a comprehensive payload that included both *in situ* and remote sensing instrumentation (figure 2) [3]. The *in situ* payload included particle and fields instruments, such as the plasma experiment (PLS), magnetometer (MAG), the cosmic ray investigation (CRS), the low-energy charged particle experiment (LECP) and the plasma wave sensors (PWS). The remote sensing payload included, the imaging experiment (ISS), the infrared spectroscopy and radiometry experiment (IRIS), the planetary radio astronomy experiment (PRA), the ultraviolet spectrometer experiment (UVS) and the photopolarimeter (PPS), as well as the radio science investigation (RSS). Together this payload, duplicated on both Voyager spacecraft, revealed early (and for the Ice Giants, the only) views of the Giant planets: Jupiter from V1 [4] and V2 [5], Saturn from V1 [6] and V2 [7], Uranus from V2 [8] and Neptune from V2 [9]. V2 also put the solar system into new perspective by capturing the solar system's family portrait and the famous ‘Pale Blue Dot’ image of Earth (figure 3).

Because V1 and V2 planned close flybys of Titan at Saturn and Triton at Neptune, respectively, they left the ecliptic plane as they careened beyond their final planetary targets: V1's trajectory bent northward (or above) the ecliptic and V2 headed southward (or below). Since both Voyager spacecraft were already outfitted with the necessary *in situ* instrumentation, these diverging trajectories presented a unique opportunity for the Heliophysics and Astrophysics communities: a chance to sample the outer limits of the solar system and its interface with the interstellar medium for the first time and from two spatially removed vantage points. Thus, on 1 January 1990, the Voyager Mission ended and the Voyager Interstellar Mission (VIM) officially began [10]. Because of the changed nature of the mission, only seven of the original 11 instruments were kept powered on: all of the particles and fields instruments, as well as the PRA and UVS. These intrepid explorers yet again boldly went where no spacecraft had gone before, becoming humankind's first interstellar explorers and providing our first (and to-date only) *in situ* observations of our solar system's termination shock [11], heliopause, heliosheath [12] and LISM [13].

Other missions have made similar transitions beyond their original mission objectives and gone on to contribute significant additional cross-disciplinary science in addition to their primary missions. The lunar reconnaissance orbiter (LRO) was conceived as part of NASA's New Vision for Space Exploration [14]. The mission launched in 2009 with an initial 1-year Exploration Mission focused on supporting the expansion of human exploration throughout the solar system and under the responsibility of the directorate now known as the Human Exploration and Operations Mission Directorate [15]. After completion of this Exploration Mission, responsibility for LRO was transferred to SMD's Planetary Science Division, where it has provided a wealth of lunar science [16]. Similarly, the Deep Impact mission to comet Tempel 1 (9P/Tempel) [17] first completed its prime mission of achieving and observing a hypervelocity impact with a comet [18] and then found a second life as the EPOXI mission, encountering comet Hartley 2 (103P/Hartley 2) [19] and exploring exoplanets [20]. Finally, the Wide-field Infrared Survey Explorer (WISE) mission was an astrophysics mission originally intended to complete a mid-infrared survey of the entire sky [21], but has since provided a wealth of observations of asteroids and near-Earth objects as the NEOWISE mission [22].

A notable example of fortuitous cross-disciplinary science comes in the form of energetic neutral atom imaging—a technique that has quite recently been used, with remarkable results, to map the outermost edges of our Sun's astrosphere. The Interstellar Boundary Explorer (IBEX) mission was launched in 2008 with the goal to map the heliospheric boundary to better understand the nature of the solar system's interaction with the LISM and provide global context to the localized *in situ* measurements from the VIM [23]; though funded by the Heliophysics Division, the science goals of IBEX are cross-disciplinary and of interest to both the Heliophysics and Astrophysics communities.

**Figure 4.**
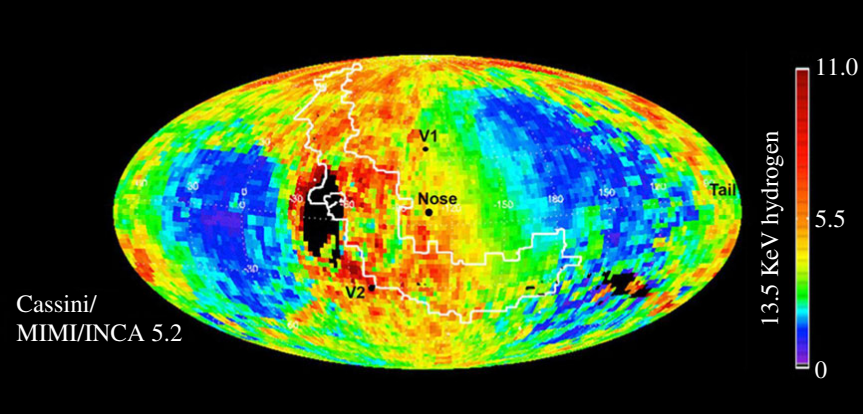
Though not initially part of its proposed objectives, the Cassini/INCA instrument was able to image the heliospheric boundary during the cruise to Saturn. These maps suggest a closed, ‘bubble-like’ shape to the heliosphere, which contrast with the conclusions from the IBEX missions; the issue remains hotly debated in the heliophysics community today. Image credit: NASA/JPL/JHUAPL. (Online version in colour.)

Soon after the launch of IBEX, further heliospheric imaging contributions came from an unlikely source: the Ion and Neutral Camera (INCA) [24] on the Cassini spacecraft en route to Saturn [25]. INCA's initial science objective was to ‘[d]etermine the global configuration and dynamics of hot plasma in the magnetosphere of Saturn through energetic neutral particle imaging of ring current, radiation belts and neutral clouds' [24]. However, during its cruise to Saturn from 2003 to 2009 INCA obtained images of the heliospheric boundary at energies not covered by the IBEX instrumentation (figure 4) [26]. Beyond providing an additional dataset, the Cassini/INCA observations provided evidence that the heliosphere is closed (i.e. a ‘bubble’) [26,27], which directly contradicted the conclusions from the IBEX observations that the heliosphere is open (i.e. has a comet-like tail) [28,29]. The debate between these two models rages on in the Heliophysics community, with hopes that it will be resolved by the upcoming Interstellar Mapping and Acceleration Probe (IMAP) mission, a Heliophysics Division-funded mission that carries updated versions of both the IBEX and Cassini/INCA instruments [30].

NASA missions have demonstrated many examples of cross-Divisional observations over the years. Cassini flybys of both Venus and Earth in 1999 [31] provided unique measurements of these planets, including the first detection of thermal emission from the Venusian surface at 0.85 and 0.9 µm [32], the escape of energetic neutral atoms from Venus' atmosphere [33], resolution of a low energy beam in the plasmasheet [34] and potential measurements of Earth's magnetotail as far away as 6000 *R_E_* [35]. Most recently, the Parker Solar Probe mission [36] completed several of its planned flybys of Venus as it dives closer into the Sun's corona [37].

**Figure 5.**
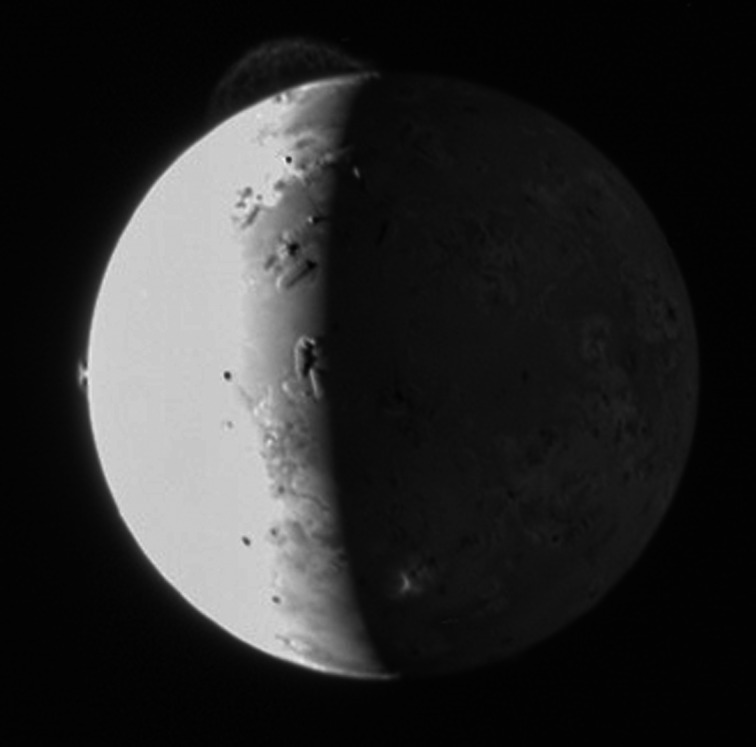
Flybys not only provide beneficial gravity assists but also additional scientific opportunities at secondary targets that can provide unexpected results, like this image of Tvashtar erupting on Io captured by New Horizons flyby of Jupiter while en route to Pluto. Image credit: NASA/JHUAPL/SwRI.

Several missions have made use of Jupiter gravity assists and in doing so, provided additional measurements of the Jovian system. For instance, the Ulysses (solar physics mission) flyby of Jupiter accessed previously un-investigated regions of the planet's magnetosphere, including higher magnetospheric latitudes, the dusk sector and inner magnetosphere [38]. Similarly, New Horizons [39] flyby flew down Jupiter's enormous (extending all the way to Saturn's orbit) magnetotail and observed escaping plasmoids [40]. Both Ulysses and New Horizons also revealed evidence of volcanic activity on Io [41,42], with the latter capturing the impressive eruption of the volcano Tvashtar (figure 5).

**Figure 6.**
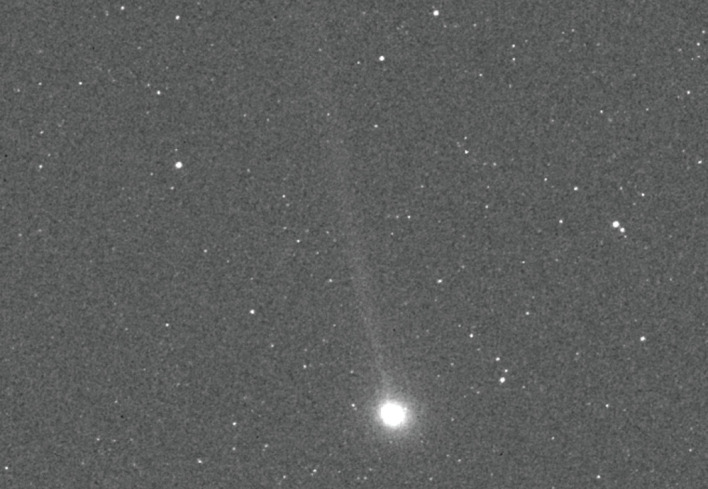
Several missions have had opportunities to make serendipitous observations of comets, like this image of 2P/Encke from the MESSENGER mission to Mercury. Image credit: NASA/JHUAPL/CIW.

Chance encounters have also provided opportunistic scientific observations for missions carrying appropriate instrumentation. For example, MESSENGER (mission to Mercury) [43] unexpectedly obtained images and spectra of comets 2P/Encke (figure 6) and C/2012 S1 (ISON). The Sun-focused Ulysses mission also captured *in situ* observations of multiple comet tails [44,45] as well as contributing to a catalogue of gamma-ray bursts from throughout the universe along with Near Earth Asteroid Rendezvous (NEAR; asteroid mission), Wind (solar wind mission) and Compton Gamma Ray Observatory (CGRO; astrophysics) [46].

Long cruise durations on the way to planetary targets, especially those in the outer solar system often provide ample opportunities to acquire additional observations, like the previously discussed heliospheric energetic neutral atom images from Cassini/INCA. Voyager and many later planetary missions (e.g. Pioneer Venus, Galileo, Cassini and MESSENGER) obtained UV measurements of interplanetary hydrogen [47] and were also able to contribute to astrophysical studies via stellar occultations [48] during cruise. Many missions, like New Horizons, have also made significant studies of solar wind [49], pick-up ionization [50] and interplanetary shock [51] evolution throughout the outer solar system.

**Figure 7.**
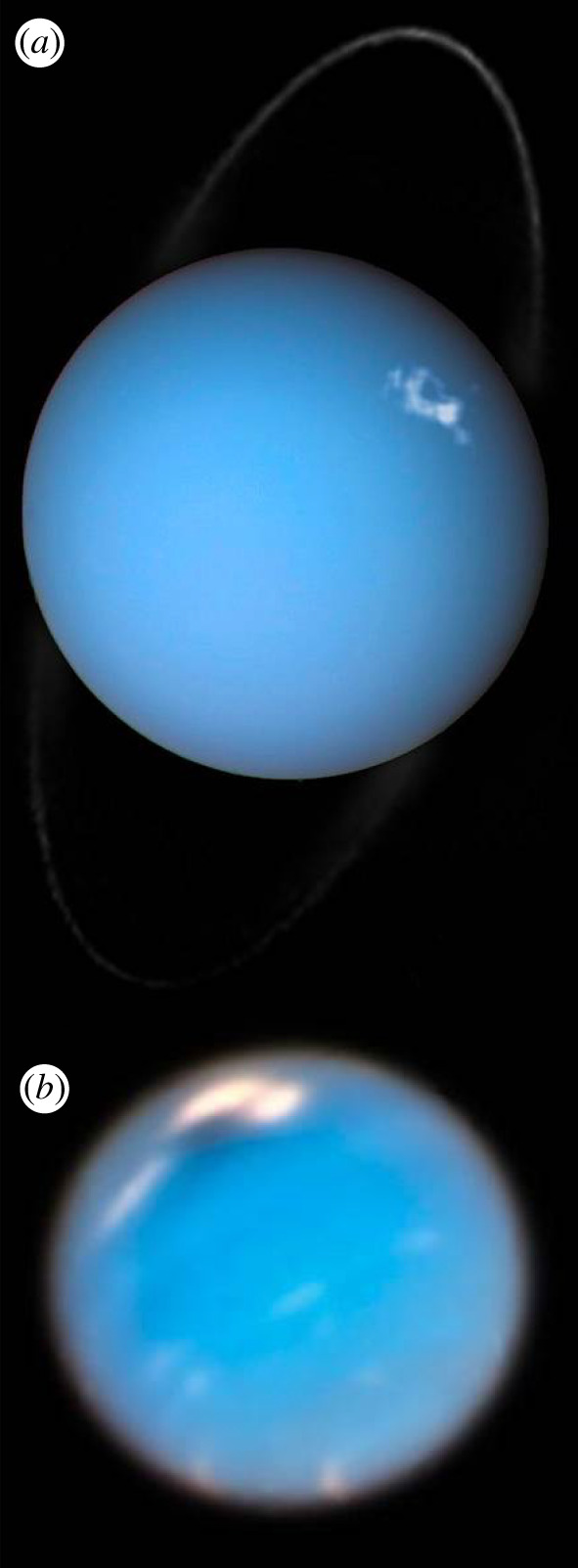
Dedicated campaigns by the Hubble Space Telescope have made significant contributions to planetary science, including capturing aurora on Uranus (*a*) and the evolution of large storms on Neptune (*b*). Image credits: ESA/Hubble & NASA, L. Lamy/Obs. De Paris; NASA/ESA/GSFC/JPL. (Online version in colour.)

Finally, Earth-based astrophysics space assets also provide invaluable cross-disciplinary measurements. For instance, the Hubble Space Telescope (HST) has provided a rich legacy of solar system observations, including giant planet images captured as part of the Outer Planets Atmospheres Legacy (OPAL) program [52]. HST images have revealed many features of the Giant planets, such as the auroral footprints from the Galilean moons at Jupiter [53], the dynamics of the rings and auroral storms at Saturn [54], auroral emissions at Uranus (figure 7*a*) [55] and the development of large storms at Neptune (figure 7*b*) [56]. HST was also able to capture the approach, impact and aftereffects of the 2009 impact of the Shoemaker-Levy 9 comet [57] and both the Spitzer Space Telescope [58,59] and Chandra X-ray Observatory [60–63] had robust programs of solar system observations.

## 3. Challenges

These recollections of the missions that transcended their scope are by no means comprehensive. While such a cogent collection of successes could make the achievement of cross-disciplinary science seem like a routine or easy occurrence, there are significant challenges to realizing such cross-disciplinary science opportunities that all of these successful cross-disciplinary missions have had to navigate. There are several approaches that NASA (and other space agencies such as ESA and JAXA) and the broad scientific community can adopt to help towards the goal of creating and successfully capitalizing on more cross-disciplinary science opportunities in the future: increasingly facilitate and encourage healthy communication both across focused scientific communities and vertically between the leadership and staff at the respective space agencies and NASA Divisions; all members of all of scientific communities are able to actively advocate for cross-disciplinary opportunities and investigations via, for example, white papers to the respective Decadal Surveys, and by proposing cross-disciplinary science investigations by leveraging the existing infrastructure for funding (e.g. technology demonstration opportunities, rideshares, missions of opportunity, etc.). Most importantly the opportunity for cross-disciplinary science needs to be *recognized* and purposefully planned, where feasible while remaining cognizant of the current fiscal realities. As our review has hopefully demonstrated, a lot of these previous opportunities were not even conceived of until after mission launch; cross-disciplinary opportunities conceived early in mission development will likely yield the strongest return on enhancing cross-disciplinary science.

## 4. Cross-disciplinary science opportunities from a future Ice Giant Mission

The Ice Giants, as the least explored and least well-understood planets in our solar system, leave a gaping hole in the completeness of our survey of potential planetary regimes. Future missions to the Ice Giants offer prime opportunities for cross-disciplinary science and can benefit from lessons learned on previous missions, such as those discussed above.

A decade-long (or longer) cruise to either Ice Giant would provide an opportunity to study the evolution of the solar wind, acceleration processes and interplanetary shocks with radial distance from the sun. Understanding the evolution and variability of these is important for understanding stars and star–planet interactions, and thus informs not only heliophysics and planetary science, but astrophysics and exoplanetary research as well. Likewise, valuable observations of interstellar and interplanetary dust throughout the outer solar system could be obtained, building on the results of New Horizons [64], Cassini [65], Ulysses [66] and others. Acquiring energetic neutral atom images of the heliospheric boundary, as Cassini did, from the outermost regions of the solar system could provide the long baselines needed to achieve useful stereoscopic imaging of targets; images from Uranus (20 AU) or Neptune (30 AU) obtained concurrently with those from Jupiter (5 AU) and/or Earth (1 AU) could provide 3D mapping of the heliospheric boundary that could perhaps provide important constraints in the debate over the shape of the heliosphere. A long cruise would also provide many years for opportunities to obtain ultraviolet spectra of interplanetary hydrogen and distant stars via occultations. Visible, infrared and radio measurements of the sun and stars could also be obtained via occultations. Imagers or spectrometers with sufficient resolution from the distant outer solar system could continue the search for new Kuiper Belt objects (as New Horizons did) and/or exoplanets. They could also perhaps observe the planets of our solar system as exoplanets would be [67] and could help further put the worlds we know into the context of the ever-expanding catalogue of known exoplanets [68]. The exploration of the Ice Giants is intrinsically beneficial to exoplanetary science because understanding all the planetary types we have access to (which may be only a small sampling of the diversity of exoplanets that exist) helps to bound the characteristics of exoplanetary systems.

Future missions to the Ice Giants also provide opportunities to investigate additional targets, like Cassini, New Horizons, Ulysses and other predecessors, these missions would likely provide a wealth of information from measurements obtained while performing a flyby to obtain a gravity assist at another planet (likely Jupiter, Earth, Venus and/or Saturn). Those observations may be even more advantageous if timelines align such that they happen to occur simultaneously with other missions at the same target, e.g. with Europa Clipper or JUICE in the Jovian system. Such extended solar system trajectories could also provide chance encounters with comets, like MESSENGER and Ulysses experienced.

While such cross-disciplinary objectives could largely be met with instruments that already have high value for Ice Giant exploration [57–59], the opportunity to augment the baseline payload with additional instruments (perhaps with funding from other Divisions or international partners) could further expand the science return of future missions.

## 5. Conclusion

Robotic space exploration, especially to the outer solar system, is by its very nature difficult and expensive. As such, it behoves the entire space science community to work collaboratively to maximize the scientific return of missions, regardless of the primary discipline or funding source. NASA and partner international space agencies have consistently navigated these challenges and provided many lessons, both positive and cautionary, that can be learned from previous missions that have successfully bridged traditional disciplinary boundaries.

In particular, future missions to the Ice Giants at the outer reaches of the solar system can provide many opportunities for cross-disciplinary science, especially if they are purposefully planned for early on in the development of the missions. In particular, long interplanetary cruise phases and opportunities to access infrequently visited regions of the solar system beyond 10 AU should not be squandered. *In situ* particle and fields measurements advance our understanding of the evolution of the solar wind throughout the solar system, and remote sensing instruments provide unique vantage points of the heliosphere and the planets within it. Our future missions to the Ice Giants not only address outstanding questions about planetary and exoplanetary systems but also offer the chance to achieve even more far-reaching science objectives if we are willing to strive for them.

## Data accessibility

This article has no additional data.
